# Poly[[bis­(dimethyl­formamide)[μ_7_-5,5′-(methyl­enedi­oxy)diisophthalato]dizinc] dimethyl­formamide monosolvate]

**DOI:** 10.1107/S1600536811037056

**Published:** 2011-09-20

**Authors:** Chuan-Qiang Li, Wen-Ge Qiu, Hong He

**Affiliations:** a College of Environmental and Energy Engineering, Beijing University of Technology, Beijing 100124, People’s Republic of China

## Abstract

In the crystal structure of the title coordination polymer, {[Zn_2_(C_17_H_8_O_10_)(C_3_H_7_NO)_2_]·C_3_H_7_NO}_*n*_, the mol­ecular build­ing block (MBB), *viz.* {Zn_2_(CO_2_)_4_(C_3_H_7_NO)_2_}, comprises two zinc atoms, each bridged by three carboxyl­ate groups. These two Zn atoms exhibit different coordination environments: a distorted coordination intermediate between trigonal–pyramidal, and square–pyramidal formed by the two coordinated dimethyl­formamide mol­ecules and three carboxylate groups, and a distorted tetra­hedral coordination defined by carboxy­late groups of which three are bidentate bridging and the fourth is a monodentate ligand. Thus, each ligand connects four MBBs, forming the three-dimensional polymer.

## Related literature

For the use of flexible multicarboxyl­ate ligands as building blocks in the assembly of coordination frameworks, see: Kim *et al.* (2004 ▶); Zhu *et al.* (2005 ▶); Hawxwell *et al.* (2007 ▶). For the synthesis, see: Karmakar & Goldberg (2010 ▶). 
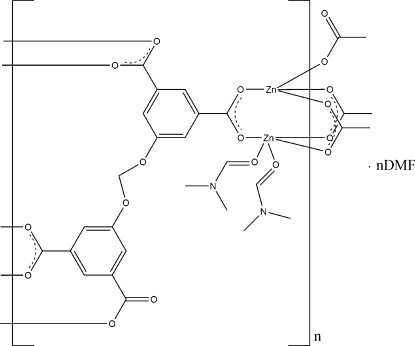


## Experimental

### 

#### Crystal data

[Zn_2_(C_17_H_8_O_10_)(C_3_H_7_NO)_2_]·C_3_H_7_NO
*M* *_r_* = 722.26
Orthorhombic, 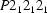

*a* = 11.2562 (6) Å
*b* = 13.1744 (7) Å
*c* = 20.3965 (12) Å
*V* = 3024.7 (3) Å^3^
*Z* = 4
Mo *K*α radiation
μ = 1.66 mm^−1^
*T* = 173 K
0.15 × 0.14 × 0.13 mm

#### Data collection

Bruker APEXII CCD diffractometer
Absorption correction: multi-scan (*SADABS*; Bruker 2008 ▶) *T*_min_ = 0.789, *T*_max_ = 0.814
15188 measured reflections
5261 independent reflections
5131 reflections with *I* > 2σ(*I*)
*R*_int_ = 0.022

#### Refinement

*R*[*F* ^2^ > 2σ(*F* ^2^)] = 0.026
*wR*(*F* ^2^) = 0.064
*S* = 1.07
5261 reflections
403 parameters
7 restraints
H-atom parameters constrained
Δρ_max_ = 1.02 e Å^−3^
Δρ_min_ = −0.51 e Å^−3^
Absolute structure: Flack (1983 ▶), 2298 Friedel pairs
Flack parameter: 0.010 (9)

### 

Data collection: *APEX2* (Bruker, 2008 ▶); cell refinement: *SAINT* (Bruker, 2008 ▶); data reduction: *SAINT*; program(s) used to solve structure: *SHELXS97* (Sheldrick, 2008) ▶; program(s) used to refine structure: *SHELXL97* (Sheldrick, 2008) ▶; molecular graphics: *SHELXTL* (Sheldrick, 2008) ▶; software used to prepare material for publication: *SHELXTL* ▶.

## Supplementary Material

Crystal structure: contains datablock(s) global, I. DOI: 10.1107/S1600536811037056/kp2331sup1.cif

Structure factors: contains datablock(s) I. DOI: 10.1107/S1600536811037056/kp2331Isup2.hkl

Additional supplementary materials:  crystallographic information; 3D view; checkCIF report

## Figures and Tables

**Table 1:** Selected bond lengths (Å)

Zn1—O10^i^	1.9779 (19)
Zn1—O4^ii^	2.010 (2)
Zn1—O11	2.030 (2)
Zn1—O2	2.052 (2)
Zn1—O12	2.073 (2)
Zn2—O8^iii^	1.953 (2)
Zn2—O9^i^	1.966 (2)
Zn2—O3^ii^	1.967 (2)
Zn2—O1	1.986 (2)

Symmetry codes: (i) 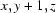; (ii) 
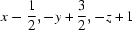
; (iii) 
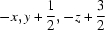
.

**Fig. 1.**
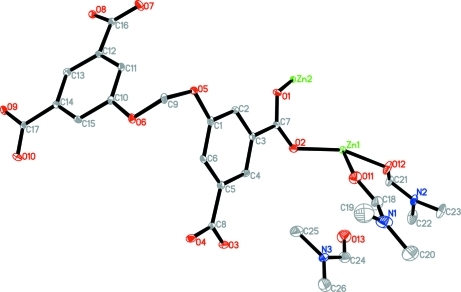
Crystal structure of compound(1) with labeling and displacement ellipsoids drawn at the 30% probability level; the hydrogen atoms and DMF molecules have been omitted for clarity.

**Fig. 2.**
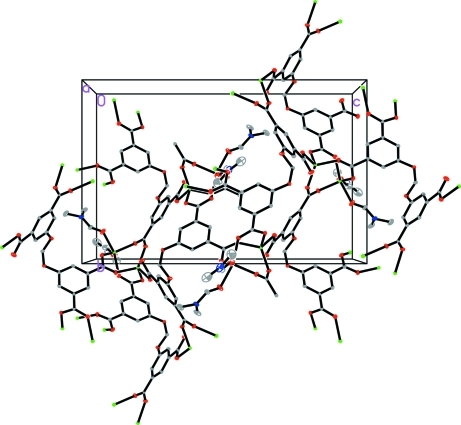
The perspective view of the polymeric structure. The hydrogen atoms and solvent moleculs have been omitted for clarity.

## supplementary crystallographic information

### Comment

The use of flexible multicarboxylate ligands as building blocks in the assembly of coordination frameworks has been an attractive strategy because some conformational freedom of the ligand molecules may offer various possibilities for release of the steric strain imposed by the metal-ligand association and relaxation of the network architecture (Kim *et al.* 2004; Zhu *et al.* 2005). Herein we chose a flexible multicarboxylate ligand, 1,1-bis-[3,5-bis(carboxy) phenoxy]methane, reacted with zinc nitrate affording a new three-dimensional coordination polymer, namely the title compound, (I), [Zn(ii)_2_(*L*)(DMF)_3_]_n_, where *L*=1,1-bis-[3,5-bis(carboxylato) phenoxy]methane, DMF=*N*,*N*-dimethyl formamide.

The molecular building blocks (MBBs) of the compound (I) comprise two zinc centres, in which the two Zn atoms are five-coordinated and four-coordinated respectively. The different coordination environments in the dinuclear zinc cluster (Fig. 1, Table 1) reveal the Zn1 centre coordinated by five oxygen atoms from  three *L* ligands and two DMF molecules, while the Zn2 centre is coordinated by four oxygen atoms from four *L* ligands. A non-coordinated solvent molecule of DMF occupies the interstitial voids within the framework.

### Experimental

The tetracarboxylic ligand, H_4_L was synthesised using the literature method of Karmakar (2010). A mixture of zinc nitrate hexahydrate (0.02 mmol, 6 mg) and the H_4_L ligand (0.01 mmol, 3.8 mg) in DMF (0.8 mL) was placed in a 5 mL sealed glass vial, and heated at 358 K for 72 h. Colourless crystals of the title compound were obtained after cooling to room temperature (yield of 52%, based on H_4_L).

### Refinement

Refinement of F^2^ against ALL reflections. The weighted *R*-factor *wR* and goodness of fit S are based on F^2^, conventional *R*-factors *R* are based on F, with F set to zero for negative F^2^. The threshold expression of F^2^ > 2sigma(F^2^) is used only for calculating *R*-factors(gt) *etc*. and is not relevant to the choice of reflections for refinement. *R*-factors based on F^2^ are statistically about twice as large as those based on F, and R– factors based on ALL data will be even larger.

### Crystal data

**Table:**

[Zn_2_(C_17_H_8_O_10_)(C_3_H_7_NO)_2_]·C_3_H_7_NO	*F*(000) = 1480
*M**_r_* = 722.26	*D*_x_ = 1.586 Mg m^−^^3^
Orthorhombic, *P*2_1_2_1_2_1_	Mo *K*α radiation, λ = 0.71073 Å
Hall symbol: P 2ac 2ab	Cell parameters from 9981 reflections
*a* = 11.2562 (6) Å	θ = 2.4–28.2°
*b* = 13.1744 (7) Å	µ = 1.66 mm^−^^1^
*c* = 20.3965 (12) Å	*T* = 173 K
*V* = 3024.7 (3) Å^3^	Block, colourless
*Z* = 4	0.15 × 0.14 × 0.13 mm

### Data collection

**Table:**

Bruker APEXII CCD diffractometer	5261 independent reflections
Radiation source: fine-focus sealed tube	5131 reflections with *I* > 2σ(*I*)
graphite	*R*_int_ = 0.022
φ and ω scans	θ_max_ = 25.0°, θ_min_ = 2.0°
Absorption correction: multi-scan (*SADABS*; Bruker 2008)	*h* = −12→13
*T*_min_ = 0.789, *T*_max_ = 0.814	*k* = −15→12
15188 measured reflections	*l* = −16→24

### Refinement

**Table:**

Refinement on *F*^2^	Secondary atom site location: difference Fourier map
Least-squares matrix: full	Hydrogen site location: inferred from neighbouring sites
*R*[*F*^2^ > 2σ(*F*^2^)] = 0.026	H-atom parameters constrained
*wR*(*F*^2^) = 0.064	*w* = 1/[σ^2^(*F*_o_^2^) + (0.0225*P*)^2^ + 2.9204*P*] where *P* = (*F*_o_^2^ + 2*F*_c_^2^)/3
*S* = 1.07	(Δ/σ)_max_ = 0.001
5261 reflections	Δρ_max_ = 1.02 e Å^−^^3^
403 parameters	Δρ_min_ = −0.51 e Å^−^^3^
7 restraints	Absolute structure: Flack (1983), 2298 Friedel pairs
Primary atom site location: structure-invariant direct methods	Flack parameter: 0.010 (9)

### Special details

**Table:**

Geometry. All e.s.d.'s (except the e.s.d. in the dihedral angle between two l.s. planes) are estimated using the full covariance matrix. The cell e.s.d.'s are taken into account individually in the estimation of e.s.d.'s in distances, angles and torsion angles; correlations between e.s.d.'s in cell parameters are only used when they are defined by crystal symmetry. An approximate (isotropic) treatment of cell e.s.d.'s is used for estimating e.s.d.'s involving l.s. planes.
Refinement. Refinement of *F*^2^ against ALL reflections. The weighted *R*-factor *wR* and goodness of fit *S* are based on *F*^2^, conventional *R*-factors *R* are based on *F*, with *F* set to zero for negative *F*^2^. The threshold expression of *F*^2^ > σ(*F*^2^) is used only for calculating *R*-factors(gt) *etc*. and is not relevant to the choice of reflections for refinement. *R*-factors based on *F*^2^ are statistically about twice as large as those based on *F*, and *R*- factors based on ALL data will be even larger.

### Fractional atomic coordinates and isotropic or equivalent isotropic displacement parameters (Å^2^)

**Table:**

	*x*	*y*	*z*	*U*_iso_*/*U*_eq_	
Zn1	0.32282 (3)	1.02153 (3)	0.532246 (16)	0.01304 (8)	
Zn2	0.11040 (3)	0.92795 (2)	0.640664 (15)	0.01034 (8)	
O1	0.25310 (18)	0.84177 (15)	0.65422 (10)	0.0142 (4)	
O2	0.3248 (2)	0.86905 (15)	0.55265 (10)	0.0186 (5)	
O3	0.55588 (18)	0.60034 (16)	0.44912 (10)	0.0173 (5)	
O4	0.6626 (2)	0.49210 (16)	0.51036 (10)	0.0226 (5)	
O5	0.48922 (18)	0.55461 (15)	0.74102 (10)	0.0141 (4)	
O6	0.51506 (17)	0.38160 (16)	0.71297 (10)	0.0142 (4)	
O7	0.13001 (19)	0.47767 (17)	0.83474 (11)	0.0232 (5)	
O8	0.00425 (18)	0.35644 (16)	0.80428 (10)	0.0157 (4)	
O9	0.14499 (16)	0.06646 (15)	0.67100 (10)	0.0151 (4)	
O10	0.32214 (19)	0.08120 (16)	0.62123 (9)	0.0166 (4)	
O11	0.5015 (2)	1.0047 (2)	0.52425 (14)	0.0353 (6)	
O12	0.3428 (2)	1.15866 (19)	0.48368 (12)	0.0306 (6)	
O13	0.3138 (3)	1.0302 (2)	0.33443 (15)	0.0499 (7)	
N1	0.6865 (3)	1.0308 (3)	0.48390 (18)	0.0495 (8)	
N2	0.3226 (3)	1.2560 (2)	0.39360 (14)	0.0247 (6)	
N3	0.4694 (3)	0.9184 (2)	0.35115 (14)	0.0322 (7)	
C1	0.4858 (3)	0.5963 (2)	0.67844 (14)	0.0138 (6)	
C2	0.4123 (3)	0.6810 (2)	0.67289 (14)	0.0137 (6)	
H2	0.3707	0.7057	0.7101	0.016*	
C3	0.4002 (3)	0.7295 (2)	0.61255 (14)	0.0137 (6)	
C4	0.4597 (3)	0.6924 (2)	0.55776 (15)	0.0146 (6)	
H4	0.4517	0.7256	0.5167	0.018*	
C5	0.5308 (3)	0.6065 (2)	0.56331 (15)	0.0141 (6)	
C6	0.5468 (3)	0.5589 (2)	0.62467 (14)	0.0126 (6)	
H6	0.5986	0.5023	0.6290	0.015*	
C7	0.3202 (3)	0.8206 (2)	0.60605 (14)	0.0130 (6)	
C8	0.5879 (2)	0.5635 (2)	0.50290 (14)	0.0149 (6)	
C9	0.5557 (3)	0.4640 (2)	0.75127 (14)	0.0148 (6)	
H9A	0.6401	0.4772	0.7407	0.018*	
H9B	0.5511	0.4452	0.7982	0.018*	
C10	0.4025 (3)	0.3430 (2)	0.72419 (13)	0.0129 (6)	
C11	0.3158 (3)	0.3889 (2)	0.76253 (14)	0.0139 (6)	
H11	0.3324	0.4504	0.7851	0.017*	
C12	0.2037 (3)	0.3435 (2)	0.76758 (14)	0.0136 (6)	
C13	0.1795 (3)	0.2524 (2)	0.73604 (13)	0.0134 (6)	
H13	0.1039	0.2212	0.7409	0.016*	
C14	0.2666 (3)	0.2068 (2)	0.69721 (14)	0.0127 (6)	
C15	0.3786 (3)	0.2523 (2)	0.69091 (14)	0.0126 (6)	
H15	0.4379	0.2218	0.6642	0.015*	
C16	0.1075 (3)	0.3978 (2)	0.80611 (13)	0.0144 (6)	
C17	0.2425 (2)	0.1110 (2)	0.66028 (13)	0.0111 (6)	
C18	0.5723 (3)	1.0484 (3)	0.4863 (2)	0.0432 (11)	
H18	0.5409	1.0978	0.4571	0.052*	
C19	0.7341 (6)	0.9558 (5)	0.5254 (3)	0.0816 (16)	
H19A	0.6697	0.9236	0.5502	0.122*	
H19B	0.7906	0.9871	0.5558	0.122*	
H19C	0.7750	0.9045	0.4990	0.122*	
C20	0.7647 (4)	1.0820 (5)	0.4373 (3)	0.0732 (17)	
H20A	0.7176	1.1271	0.4093	0.110*	
H20B	0.8049	1.0313	0.4100	0.110*	
H20C	0.8240	1.1219	0.4612	0.110*	
C21	0.2853 (3)	1.1867 (3)	0.43474 (17)	0.0240 (7)	
H21	0.2103	1.1560	0.4268	0.029*	
C22	0.2583 (4)	1.2772 (3)	0.3324 (2)	0.0437 (11)	
H22A	0.3031	1.2496	0.2952	0.066*	
H22B	0.2492	1.3507	0.3270	0.066*	
H22C	0.1796	1.2453	0.3341	0.066*	
C23	0.4417 (3)	1.2999 (3)	0.3995 (2)	0.0364 (10)	
H23A	0.4678	1.2963	0.4453	0.055*	
H23B	0.4399	1.3710	0.3853	0.055*	
H23C	0.4972	1.2617	0.3718	0.055*	
C24	0.4167 (3)	1.0000 (3)	0.32387 (19)	0.0347 (9)	
H24	0.4628	1.0381	0.2936	0.042*	
C25	0.4002 (5)	0.8543 (3)	0.3960 (2)	0.0493 (11)	
H25A	0.3157	0.8708	0.3918	0.074*	
H25B	0.4260	0.8666	0.4412	0.074*	
H25C	0.4128	0.7827	0.3849	0.074*	
C26	0.5865 (4)	0.8865 (4)	0.3345 (2)	0.0451 (10)	
H26A	0.6201	0.9331	0.3019	0.068*	
H26B	0.5837	0.8178	0.3163	0.068*	
H26C	0.6364	0.8868	0.3739	0.068*	

### Atomic displacement parameters (Å^2^)

**Table:**

	*U*^11^	*U*^22^	*U*^33^	*U*^12^	*U*^13^	*U*^23^
Zn1	0.01357 (16)	0.01255 (16)	0.01301 (16)	−0.00049 (14)	0.00072 (13)	−0.00015 (14)
Zn2	0.01203 (15)	0.00882 (15)	0.01017 (15)	−0.00059 (13)	0.00052 (13)	−0.00001 (13)
O1	0.0166 (10)	0.0147 (10)	0.0114 (10)	0.0036 (8)	0.0003 (8)	−0.0023 (8)
O2	0.0252 (11)	0.0154 (11)	0.0153 (11)	0.0040 (10)	0.0024 (9)	0.0010 (8)
O3	0.0182 (10)	0.0207 (12)	0.0129 (10)	0.0038 (9)	0.0020 (9)	0.0006 (9)
O4	0.0282 (12)	0.0181 (12)	0.0215 (11)	0.0101 (10)	0.0129 (9)	0.0061 (9)
O5	0.0210 (10)	0.0110 (11)	0.0102 (10)	0.0011 (8)	0.0001 (8)	0.0012 (8)
O6	0.0106 (10)	0.0111 (10)	0.0209 (11)	−0.0012 (8)	0.0009 (8)	−0.0023 (9)
O7	0.0230 (11)	0.0171 (11)	0.0296 (12)	0.0006 (10)	0.0042 (9)	−0.0094 (11)
O8	0.0152 (10)	0.0145 (11)	0.0175 (11)	0.0009 (9)	0.0041 (8)	−0.0011 (9)
O9	0.0145 (10)	0.0104 (10)	0.0203 (10)	−0.0009 (8)	0.0005 (8)	−0.0030 (9)
O10	0.0182 (10)	0.0173 (10)	0.0143 (10)	0.0026 (10)	0.0014 (9)	−0.0050 (8)
O11	0.0201 (12)	0.0384 (16)	0.0474 (16)	0.0026 (11)	0.0074 (11)	0.0003 (13)
O12	0.0300 (13)	0.0318 (13)	0.0301 (13)	−0.0156 (11)	−0.0114 (11)	0.0180 (11)
O13	0.0426 (16)	0.0524 (18)	0.0548 (18)	0.0067 (16)	0.0051 (15)	0.0042 (15)
N1	0.0242 (16)	0.074 (2)	0.050 (2)	−0.0051 (17)	0.0078 (15)	−0.0203 (16)
N2	0.0297 (15)	0.0177 (14)	0.0266 (15)	−0.0068 (13)	−0.0030 (13)	0.0092 (12)
N3	0.0499 (19)	0.0250 (16)	0.0216 (15)	−0.0043 (14)	0.0147 (14)	0.0027 (13)
C1	0.0152 (14)	0.0117 (15)	0.0145 (15)	−0.0040 (11)	−0.0043 (12)	0.0008 (11)
C2	0.0153 (15)	0.0124 (14)	0.0134 (14)	−0.0011 (12)	0.0024 (12)	−0.0015 (11)
C3	0.0150 (14)	0.0107 (14)	0.0154 (14)	−0.0014 (12)	0.0012 (12)	−0.0002 (11)
C4	0.0156 (14)	0.0165 (15)	0.0117 (13)	0.0011 (12)	0.0003 (12)	0.0025 (12)
C5	0.0114 (14)	0.0139 (15)	0.0172 (15)	−0.0003 (11)	0.0016 (12)	−0.0001 (12)
C6	0.0130 (13)	0.0091 (14)	0.0157 (15)	0.0006 (11)	0.0001 (11)	−0.0019 (11)
C7	0.0135 (14)	0.0115 (14)	0.0140 (14)	0.0004 (12)	−0.0003 (13)	−0.0005 (11)
C8	0.0136 (14)	0.0130 (14)	0.0181 (15)	−0.0019 (13)	0.0064 (12)	0.0006 (12)
C9	0.0198 (14)	0.0093 (15)	0.0154 (14)	0.0003 (12)	−0.0062 (12)	0.0026 (12)
C10	0.0135 (14)	0.0110 (14)	0.0142 (14)	0.0002 (12)	−0.0021 (12)	0.0032 (11)
C11	0.0170 (15)	0.0103 (13)	0.0145 (14)	−0.0026 (12)	0.0000 (12)	−0.0004 (11)
C12	0.0170 (15)	0.0126 (14)	0.0111 (14)	0.0029 (12)	0.0012 (11)	0.0008 (11)
C13	0.0126 (14)	0.0144 (14)	0.0131 (14)	0.0002 (12)	−0.0009 (12)	0.0011 (11)
C14	0.0167 (14)	0.0115 (14)	0.0100 (14)	0.0031 (12)	−0.0020 (12)	0.0022 (12)
C15	0.0140 (14)	0.0124 (14)	0.0113 (13)	0.0038 (12)	−0.0003 (12)	−0.0014 (11)
C16	0.0179 (14)	0.0137 (14)	0.0116 (13)	0.0016 (12)	−0.0007 (13)	0.0003 (11)
C17	0.0145 (15)	0.0087 (14)	0.0101 (13)	0.0022 (11)	−0.0023 (11)	0.0030 (11)
C18	0.0231 (19)	0.036 (2)	0.071 (3)	−0.0021 (16)	0.0152 (19)	−0.006 (2)
C19	0.0789 (18)	0.0864 (18)	0.0795 (18)	0.0028 (10)	0.0012 (10)	0.0001 (10)
C20	0.037 (2)	0.086 (4)	0.097 (4)	−0.020 (3)	0.032 (3)	−0.026 (4)
C21	0.0234 (17)	0.0222 (17)	0.0265 (18)	−0.0087 (14)	−0.0017 (14)	0.0063 (15)
C22	0.054 (3)	0.037 (2)	0.040 (2)	−0.015 (2)	−0.021 (2)	0.0180 (19)
C23	0.030 (2)	0.030 (2)	0.050 (2)	−0.0078 (16)	−0.0021 (18)	0.0211 (18)
C24	0.037 (2)	0.035 (2)	0.032 (2)	0.0023 (16)	0.0013 (16)	0.0007 (16)
C25	0.071 (3)	0.043 (2)	0.034 (2)	0.003 (2)	0.017 (2)	0.0046 (19)
C26	0.032 (2)	0.042 (2)	0.061 (3)	−0.0001 (18)	0.003 (2)	−0.010 (2)

### Geometric parameters (Å, °)

**Table:**

Zn1—O10^i^	1.9779 (19)	C3—C4	1.391 (4)
Zn1—O4^ii^	2.010 (2)	C3—C7	1.506 (4)
Zn1—O11	2.030 (2)	C4—C5	1.390 (4)
Zn1—O2	2.052 (2)	C4—H4	0.9500
Zn1—O12	2.073 (2)	C5—C6	1.411 (4)
Zn2—O8^iii^	1.953 (2)	C5—C8	1.501 (4)
Zn2—O9^i^	1.966 (2)	C6—H6	0.9500
Zn2—O3^ii^	1.967 (2)	C9—H9A	0.9900
Zn2—O1	1.986 (2)	C9—H9B	0.9900
O1—C7	1.270 (3)	C10—C11	1.389 (4)
O2—C7	1.263 (3)	C10—C15	1.400 (4)
O3—C8	1.253 (4)	C11—C12	1.400 (4)
O3—Zn2^iv^	1.967 (2)	C11—H11	0.9500
O4—C8	1.270 (4)	C12—C13	1.389 (4)
O4—Zn1^iv^	2.010 (2)	C12—C16	1.517 (4)
O5—C1	1.390 (3)	C13—C14	1.396 (4)
O5—C9	1.424 (4)	C13—H13	0.9500
O6—C10	1.384 (3)	C14—C15	1.402 (4)
O6—C9	1.414 (4)	C14—C17	1.496 (4)
O7—C16	1.230 (4)	C15—H15	0.9500
O8—C16	1.284 (4)	C18—H18	0.9500
O8—Zn2^v^	1.953 (2)	C19—H19A	0.9800
O9—C17	1.263 (3)	C19—H19B	0.9800
O9—Zn2^vi^	1.966 (2)	C19—H19C	0.9800
O10—C17	1.262 (3)	C20—H20A	0.9800
O10—Zn1^vi^	1.9779 (19)	C20—H20B	0.9800
O11—C18	1.252 (5)	C20—H20C	0.9800
O12—C21	1.245 (4)	C21—H21	0.9500
O13—C24	1.244 (5)	C22—H22A	0.9800
N1—C18	1.307 (5)	C22—H22B	0.9800
N1—C19	1.407 (7)	C22—H22C	0.9800
N1—C20	1.460 (6)	C23—H23A	0.9800
N2—C21	1.310 (4)	C23—H23B	0.9800
N2—C23	1.466 (5)	C23—H23C	0.9800
N2—C22	1.470 (5)	C24—H24	0.9500
N3—C24	1.348 (5)	C25—H25A	0.9800
N3—C26	1.424 (5)	C25—H25B	0.9800
N3—C25	1.468 (5)	C25—H25C	0.9800
C1—C6	1.385 (4)	C26—H26A	0.9800
C1—C2	1.394 (4)	C26—H26B	0.9800
C2—C3	1.393 (4)	C26—H26C	0.9800
C2—H2	0.9500		
			
O10^i^—Zn1—O4^ii^	115.39 (9)	C11—C10—C15	120.6 (3)
O10^i^—Zn1—O11	96.95 (10)	C10—C11—C12	119.3 (3)
O4^ii^—Zn1—O11	147.61 (11)	C10—C11—H11	120.4
O10^i^—Zn1—O2	101.72 (8)	C12—C11—H11	120.4
O4^ii^—Zn1—O2	90.56 (9)	C13—C12—C11	120.8 (3)
O11—Zn1—O2	84.18 (10)	C13—C12—C16	120.5 (3)
O10^i^—Zn1—O12	95.31 (10)	C11—C12—C16	118.7 (3)
O4^ii^—Zn1—O12	88.20 (9)	C12—C13—C14	119.8 (3)
O11—Zn1—O12	87.10 (10)	C12—C13—H13	120.1
O2—Zn1—O12	161.68 (9)	C14—C13—H13	120.1
O8^iii^—Zn2—O9^i^	113.43 (9)	C13—C14—C15	120.0 (3)
O8^iii^—Zn2—O3^ii^	103.75 (9)	C13—C14—C17	121.4 (3)
O9^i^—Zn2—O3^ii^	122.06 (9)	C15—C14—C17	118.5 (3)
O8^iii^—Zn2—O1	100.29 (8)	C10—C15—C14	119.5 (3)
O9^i^—Zn2—O1	109.06 (8)	C10—C15—H15	120.2
O3^ii^—Zn2—O1	105.88 (9)	C14—C15—H15	120.2
C7—O1—Zn2	119.91 (18)	O7—C16—O8	124.3 (3)
C7—O2—Zn1	131.99 (19)	O7—C16—C12	120.2 (3)
C8—O3—Zn2^iv^	130.68 (19)	O8—C16—C12	115.5 (2)
C8—O4—Zn1^iv^	127.46 (18)	O10—C17—O9	125.6 (3)
C1—O5—C9	118.7 (2)	O10—C17—C14	116.8 (3)
C10—O6—C9	119.1 (2)	O9—C17—C14	117.5 (3)
C16—O8—Zn2^v^	112.12 (18)	O11—C18—N1	124.6 (4)
C17—O9—Zn2^vi^	123.25 (18)	O11—C18—H18	117.7
C17—O10—Zn1^vi^	134.87 (19)	N1—C18—H18	117.7
C18—O11—Zn1	129.1 (3)	N1—C19—H19A	109.5
C21—O12—Zn1	125.8 (2)	N1—C19—H19B	109.5
C18—N1—C19	118.5 (5)	H19A—C19—H19B	109.5
C18—N1—C20	122.3 (5)	N1—C19—H19C	109.5
C19—N1—C20	119.1 (4)	H19A—C19—H19C	109.5
C21—N2—C23	121.0 (3)	H19B—C19—H19C	109.5
C21—N2—C22	121.3 (3)	N1—C20—H20A	109.5
C23—N2—C22	116.5 (3)	N1—C20—H20B	109.5
C24—N3—C26	122.9 (3)	H20A—C20—H20B	109.5
C24—N3—C25	118.8 (3)	N1—C20—H20C	109.5
C26—N3—C25	118.0 (4)	H20A—C20—H20C	109.5
C6—C1—O5	125.0 (3)	H20B—C20—H20C	109.5
C6—C1—C2	121.0 (3)	O12—C21—N2	123.7 (3)
O5—C1—C2	114.1 (3)	O12—C21—H21	118.2
C3—C2—C1	119.8 (3)	N2—C21—H21	118.2
C3—C2—H2	120.1	N2—C22—H22A	109.5
C1—C2—H2	120.1	N2—C22—H22B	109.5
C4—C3—C2	120.1 (3)	H22A—C22—H22B	109.5
C4—C3—C7	119.8 (3)	N2—C22—H22C	109.5
C2—C3—C7	120.1 (3)	H22A—C22—H22C	109.5
C5—C4—C3	119.8 (3)	H22B—C22—H22C	109.5
C5—C4—H4	120.1	N2—C23—H23A	109.5
C3—C4—H4	120.1	N2—C23—H23B	109.5
C4—C5—C6	120.5 (3)	H23A—C23—H23B	109.5
C4—C5—C8	119.2 (3)	N2—C23—H23C	109.5
C6—C5—C8	120.3 (3)	H23A—C23—H23C	109.5
C1—C6—C5	118.8 (3)	H23B—C23—H23C	109.5
C1—C6—H6	120.6	O13—C24—N3	126.4 (4)
C5—C6—H6	120.6	O13—C24—H24	116.8
O2—C7—O1	125.5 (3)	N3—C24—H24	116.8
O2—C7—C3	117.0 (3)	N3—C25—H25A	109.5
O1—C7—C3	117.5 (2)	N3—C25—H25B	109.5
O3—C8—O4	125.6 (3)	H25A—C25—H25B	109.5
O3—C8—C5	116.7 (2)	N3—C25—H25C	109.5
O4—C8—C5	117.7 (3)	H25A—C25—H25C	109.5
O6—C9—O5	113.1 (2)	H25B—C25—H25C	109.5
O6—C9—H9A	109.0	N3—C26—H26A	109.5
O5—C9—H9A	109.0	N3—C26—H26B	109.5
O6—C9—H9B	109.0	H26A—C26—H26B	109.5
O5—C9—H9B	109.0	N3—C26—H26C	109.5
H9A—C9—H9B	107.8	H26A—C26—H26C	109.5
O6—C10—C11	125.2 (3)	H26B—C26—H26C	109.5
O6—C10—C15	114.1 (3)		
			
O8^iii^—Zn2—O1—C7	138.9 (2)	C6—C5—C8—O3	169.7 (3)
O9^i^—Zn2—O1—C7	−101.7 (2)	C4—C5—C8—O4	172.4 (3)
O3^ii^—Zn2—O1—C7	31.3 (2)	C6—C5—C8—O4	−9.2 (4)
O10^i^—Zn1—O2—C7	3.6 (3)	C10—O6—C9—O5	−65.0 (3)
O4^ii^—Zn1—O2—C7	−112.5 (3)	C1—O5—C9—O6	−60.1 (3)
O11—Zn1—O2—C7	99.5 (3)	C9—O6—C10—C11	11.8 (4)
O12—Zn1—O2—C7	161.5 (3)	C9—O6—C10—C15	−170.6 (2)
O10^i^—Zn1—O11—C18	−113.8 (4)	O6—C10—C11—C12	177.4 (3)
O4^ii^—Zn1—O11—C18	63.2 (4)	C15—C10—C11—C12	0.0 (4)
O2—Zn1—O11—C18	145.0 (4)	C10—C11—C12—C13	1.5 (4)
O12—Zn1—O11—C18	−18.8 (4)	C10—C11—C12—C16	−176.2 (2)
O10^i^—Zn1—O12—C21	−134.1 (3)	C11—C12—C13—C14	−1.9 (4)
O4^ii^—Zn1—O12—C21	−18.7 (3)	C16—C12—C13—C14	175.7 (3)
O11—Zn1—O12—C21	129.2 (3)	C12—C13—C14—C15	0.9 (4)
O2—Zn1—O12—C21	67.6 (5)	C12—C13—C14—C17	−177.5 (3)
C9—O5—C1—C6	−3.0 (4)	O6—C10—C15—C14	−178.6 (2)
C9—O5—C1—C2	176.2 (2)	C11—C10—C15—C14	−0.9 (4)
C6—C1—C2—C3	−0.3 (4)	C13—C14—C15—C10	0.5 (4)
O5—C1—C2—C3	−179.5 (2)	C17—C14—C15—C10	179.0 (2)
C1—C2—C3—C4	1.1 (4)	Zn2^v^—O8—C16—O7	−5.5 (4)
C1—C2—C3—C7	179.5 (3)	Zn2^v^—O8—C16—C12	176.76 (18)
C2—C3—C4—C5	0.2 (4)	C13—C12—C16—O7	178.9 (3)
C7—C3—C4—C5	−178.1 (3)	C11—C12—C16—O7	−3.4 (4)
C3—C4—C5—C6	−2.4 (4)	C13—C12—C16—O8	−3.3 (4)
C3—C4—C5—C8	176.0 (3)	C11—C12—C16—O8	174.4 (3)
O5—C1—C6—C5	177.3 (3)	Zn1^vi^—O10—C17—O9	41.3 (4)
C2—C1—C6—C5	−1.8 (4)	Zn1^vi^—O10—C17—C14	−139.2 (2)
C4—C5—C6—C1	3.2 (4)	Zn2^vi^—O9—C17—O10	11.7 (4)
C8—C5—C6—C1	−175.2 (3)	Zn2^vi^—O9—C17—C14	−167.89 (18)
Zn1—O2—C7—O1	41.7 (5)	C13—C14—C17—O10	172.8 (3)
Zn1—O2—C7—C3	−139.1 (2)	C15—C14—C17—O10	−5.6 (4)
Zn2—O1—C7—O2	19.6 (4)	C13—C14—C17—O9	−7.6 (4)
Zn2—O1—C7—C3	−159.64 (19)	C15—C14—C17—O9	174.0 (2)
C4—C3—C7—O2	−10.6 (4)	Zn1—O11—C18—N1	−178.6 (3)
C2—C3—C7—O2	171.0 (3)	C19—N1—C18—O11	1.8 (7)
C4—C3—C7—O1	168.7 (3)	C20—N1—C18—O11	178.2 (4)
C2—C3—C7—O1	−9.7 (4)	Zn1—O12—C21—N2	−157.8 (3)
Zn2^iv^—O3—C8—O4	−4.0 (5)	C23—N2—C21—O12	5.5 (6)
Zn2^iv^—O3—C8—C5	177.16 (19)	C22—N2—C21—O12	172.3 (4)
Zn1^iv^—O4—C8—O3	47.0 (4)	C26—N3—C24—O13	177.5 (4)
Zn1^iv^—O4—C8—C5	−134.2 (2)	C25—N3—C24—O13	2.8 (6)
C4—C5—C8—O3	−8.7 (4)		

Symmetry codes: (i) *x*, *y*+1, *z*; (ii) *x*−1/2, −*y*+3/2, −*z*+1; (iii) −*x*, *y*+1/2, −*z*+3/2; (iv) *x*+1/2, −*y*+3/2, −*z*+1; (v) −*x*, *y*−1/2, −*z*+3/2; (vi) *x*, *y*−1, *z*.
